# Downregulation of long non-coding RNA MAFG-AS1 represses tumorigenesis of colorectal cancer cells through the microRNA-149-3p-dependent inhibition of HOXB8

**DOI:** 10.1186/s12935-020-01485-4

**Published:** 2020-10-19

**Authors:** Zhiyan Ruan, Hongling Deng, Minhua Liang, Zhe Xu, Manxiang Lai, Hong Ren, Xiangliang Deng, Xinguo Su

**Affiliations:** 1 School of Pharmacy, Guangdong Province, Guangdong Food & Drug Vocational College, No. 321, Longdong North Road, Tianhe District, Guangzhou, 510520 P.R. China; 2 School of Chinese Medicine, Guangdong Pharmaceutical University, No. 280, East Ring Road, Guangzhou University Town, Guangzhou, 510006 P.R. China

**Keywords:** Long non-coding RNA MAFG-AS1, microRNA-149-3p, homeobox B8, colorectal cancer, biological characteristics

## Abstract

**Background:**

Colorectal cancer (CRC) is considered as the second common death-induced cancer. More recently, association of long non-coding RNAs (lncRNAs) with CRC has been extensively investigated. Therefore, the present study was performed to determine whether lncRNA MAF BZIP Transcription Factor G Antisense RNA 1 (MAFG-AS1) could regulate biological activities of CRC cells and unravel the underlying mechanisms.

**Methods:**

CRC and corresponding adjacent tissues were collected to determine the expression of lncRNA MAFG-AS1, microRNA-149-3p (miR-149-3p) and homeobox B8 (HOXB8) by RT-qPCR. Dual luciferase reporter gene assay was used to explore the targeting relationship between miR-149-3p and lncRNA MAFG-AS1 and between miR-149-3p and HOXB8, followed by RNA immunoprecipitation for verification. Migration, proliferation, invasion, and apoptosis of HCT116 and LoVo cells were examined when lncRNA MAFG-AS1 was silenced or miR-149-3p was overexpressed. Furthermore, tumorigenicity of HCT116 and LoVo cells was measured in vivo by tumor xenograft in nude mice.

**Results:**

LncRNA MAFG-AS1 and HOXB8 were found to be highly expressed in CRC tissues and cells, while miR-149-3p was under-expressed. LncRNA MAFG-AS1 negatively regulated miR-149-3p while miR-149-3p downregulated HOXB8. In addition, lncRNA MAFG-AS1 silencing by shRNA or miR-149-3p upregulation by mimic suppressed the migration, proliferation, invasion and tumorigenesis but promoted the apoptosis of HCT116 and LoVo cells.

**Conclusion:**

Taken together, lncRNA MAFG-AS1 downregulation inhibits the malignant behaviors of CRC cells by upregulating miR-149-3p and downregulating HOXB8, providing a potential therapeutic target for CRC treatment.

## Background

Colorectal cancer (CRC) is regarded as the third most common malignant cancers and the second leading cause of cancer-related death across the world, resulting in approximately 881,000 deaths in 2018 [1]. Despite of the advances made for diagnosis at the early stage, patients usually are detected with the presence of metastasis in the lung, liver and peritoneum when diagnosed as CRC [2]. In recent years, the incidence of CRC in young patients has been reported to be on an upward trend, highlighting an urgent need to develop novel biomarker and therapeutic strategy for CRC [3, 4].

Long non-coding RNAs (lncRNAs), a group of non-coding RNA longer than 200 nucleotides, have attracted research attention considering their roles as oncogenes or anti-tumor genes in the pathogenesis of CRC for diagnosis methods and treatment modalities [5, 6]. LncRNA MAF BZIP Transcription Factor G Antisense RNA 1 (MAFG-AS1) has been reported to function in multiple types of cancer, including hepatocellular carcinoma, lung adenocarcinoma, breast cancer, and non-small cell lung cancer through interplay with different microRNAs (miRNAs) [7–10]. To our best knowledge, there is only one report discussing the action of lncRNA MAFG-AS1 in CRC, where Cui et al*.* have found that MAFG-AS1 activates NDUFA4 through miR-147b to promote the progression of CRC [11].

The lncRNA-miRNA regulatory paradigms have emerged with the potential to regulate gene expression profile implicated in diverse cellular procedures, ranging from cell proliferation and differentiation to cell apoptosis [12]. A previous study has revealed has-miR-149-3p as a possible miRNA involved in the lncRNA-miRNA-mRNA axis in CRC [13]. In addition, miR-149-3p has been indicated to play an important role in chemotherapeutic effects of fluorouracil in CRC cells [14]. Importantly, miR-149-3p has been elucidated to be targeted by lncRNA prostate cancer-associated ncRNA transcripts 1 (PCAT-1) to mediate CRC cell proliferation, migration, invasion and apoptosis [15]. Bioinformatics prediction of our study revealed a specific binding site between lncRNA MAFG-AS1 and miR-149-3p. Besides, homeobox B8 (HOXB8) was predicted as a possible target gene of miR-149-3p. The therapeutic significance of HOXB8 knockdown in CRC has also been documented both in vitro and in vivo [16, 17].

Therefore, the current investigation was initiated to provide further solid evidences regarding the potential function of lncRNA MAFG-AS1 and the crosstalk between lncRNA MAFG-AS1 and miR-149-3p in the progression of CRC. We also endeavored to explore the potential effects of the lncRNA MAFG-AS1/miR-149-3p/HOXB8 axis on the invasion, migration, proliferation and apoptosis of CRC cells, which might provide some references for the treatment of CRC in the very near future.

## Materials and methods

### Ethics statement

The study was approved by the Ethics Committee of Guangdong Pharmaceutical University (approval code: GDPULAC2018125; approval date: 22 October 2018) and consents were obtained from all participants prior to our study. Animal experiments were carried out in line with the licensing agreement of the Animal Experiment Committee of Guangdong Pharmaceutical University (approval code: GDPULAC2018125; approval date: 22 October 2018). There was no animal cruelty in the experiment, and all involved animals were properly dealt with after the experiment.

### Study subjects

In total, 30 cases (13 males, 17 females, age: 24–52 years, mean age: 42.17 ± 4.26 years) of CRC tumor tissues and corresponding adjacent tissues were collected after diagnosis by biopsy. No patients were treated by radiotherapy or chemotherapy before the surgery.

### Cell treatment

Human CRC cell lines HCT116, SW1116, SW480 and LoVo were purchased from Chinese Academy of Sciences (Shanghai, China) and stored in Dulbecco’s modified eagle medium (DMEM) containing 10% fetal bovine serum (FBS) (Gibco, Carlsbad, CA, USA). Normal colon epithelial cell line NCM460 was cultured in DMEM: F12 (Gibco) supplemented with 10% FBS at 37 °C with 5% CO_2_.

Briefly, 24 h before transfection, HCT116 and LoVo cells were seeded in a 6-well plate. According to the manual of lipofectamine 2000 (11668-019, Invitrogen, NY, CA, USA) [11], cells at the logarithmic growth phase with a confluence of 30–50% were transduced with different plasmids, including blank plasmid as the shRNA-negative control (sh-NC, GenePharma Corporation, Suzhou, China), shRNA targeting MAFG-AS1 (sh-MAFG-AS1, GenePharma Corporation), mimic NC, miR-149-3p mimic, sh-HOXB8, empty pcDNA3.1 (overexpression [oe]-NC, Shanghai Sangon Biotech Co., Ltd., Shanghai, China), pcDNA3.1-HOXB8 (oe-HOXB8, Shanghai Sangon Biotech Co., Ltd.), sh-MAFG-AS1 + oe-NC, sh-MAFG-AS1 + oe-HOXB8, sh-MAFG-AS1 + inhibitor NC, or sh-MAFG-AS1 + miR-149-3p inhibitor. Briefly, 100 pmol plasmids (final concentration: 50 nM) were diluted by 250 µL serum-free Opti-MEM (51985042, Gibco) and cultured at room temperature for 5 min. Meanwhile, another 5 µL lipofectamine 2000 (11668-019, Invitrogen) was diluted by 250 µL serum-free medium Opti-MEM, and incubated for 5 min at room temperature. The above two solutions were mixed and incubated at room temperature for 20 min before they were added to the cell culture well. Following 6-h culture, medium was renewed with fresh new medium. After transfection for 48 h, cell morphology was observed under a fluorescence microscope. Then, cells were harvested to extract RNA and proteins for further experiments. The sh-NC sequence was: AAUUCUCCGAACGUGUCACGU, and the sh-MAFG-AS1 sequence was: 5′-GGGCAAUUCCAACCAAGAAAC-3′ [18].

### Reverse transcription quantitative polymerase chain reaction (RT-qPCR)

Total RNA was extracted from cells after transfection using Trizol (15596026, Invitrogen) and miRNeasy Mini Kit (217004, QIAGEN, Hilden, Germany). Primers for lncRNA MAFG-AS1, miR-149-3p and HOXB8 were designed and synthesized by TaKaRa Biotechnology Co., Ltd. (Dalian, Liaoning, China) (Table 1). Then, the extracted RNA was reversely transcribed into cDNA by PrimeScript RT Kit (RR036A, TaKaRa). The reaction solution was taken for fluorescence qPCR following the instructions of the SYBR Premix Ex Taq™ II Kit (RR820A, TaKaRa). ABI7500 qPCR instrument (7500, ABI, Foster City, USA) was applied for real-time fluorescent qPCR. The relative quantitative method (2^−△△Ct^) was adopted to calculate the relative transcriptional level of miR-149-3p (normalized to U6) and to measure the relative transcriptional levels of lncRNA MAFG-AS1 and HOXB8 mRNA (normalized to glyceraldehyde-3-phosphate dehydrogenase (GAPDH) [19].

**Table 1:** Primer sequences for reverse transcription quantitative polymerase chain reaction

Gene	Sequence (5′–3′)
MAFG-AS1	Forward: 5′-CGAAGATCTCCTCACCTCCC-3′
	Reverse: 5′-TTAAAGCCGGTCGTGGAGAT-3′
miR-149-3p	Forward: 5′-GGCTCTGGCTCCGTGTCTT-3′
	Reverse: 5′-CAGTGCAGGGTCCGAGGTATT-3′
HOXB8	Forward: 5′-ACGTGCTTCTTTGTAATGACCA-3′
	Reverse: 5′-TGTAACAATTGCCCACAGCG-3′
GAPDH	Forward: 5′-TGGCCTTCCGTGTTCCTAC-3′
	Reverse: 5′-GCTCTTTCCGCACCTTCACC-3′
U6	Forward: 5′-CAAATTCGTGAAGCGTTCCATA-3′
	Reverse: 5′-AGTGCAGGGTCCGAGGTATTC-3′

*MAFG-AS1* MAF BZIP Transcription Factor G Antisense RNA 1, *miR-149-3p* microRNA-149-3p, *HOXB8* Homeobox B8, *GAPDH* glyceraldehyde-3-phosphate dehydrogenase

### Western blot assay

The total protein was extracted from cells by radio-immunoprecipitation assay (RIPA) kit (R0010, Solarbio Science & Technology Co., Ltd., Beijing, China), and the protein concentration was determined by bicinchoninic acid kit (G3522-1, GBCBIO Technologies Inc., Guangzhou, Guangdong, China). The proteins were separated by polyacrylamide gel electrophoresis, transferred onto the nitrocellulose membrane in a wet manner and blocked for 1 h at room temperature with 5% bovine serum albumin (BSA). The membrane was probed with diluted rabbit anti-human primary antibodies to HOXB8 (H00003218-M01, 1:1000, Abnova Corp., Taipei City, Taiwan, China) at 4 °C overnight and re-probed with horseradish peroxidase-labeled goat anti-rabbit secondary antibody to immunoglobulin G (IgG) (ab205718, 1:5000, Abcam Inc., Cambridge, UK). The membrane was then developed with enhanced chemiluminescence solution (ECL808-25, Biomiga, San Diego, CA, USA) at room temperature for 1 min, covered with a fresh-keeping film and exposed to X ray (36209ES01, Shanghai Qcbio Science & Technologies Co., Ltd., Shanghai, China). The ratio of the gray value of the target band to that of GAPDH (ab8245, 1:1000, Abcam) was taken as the relative expression level of the protein to be tested [11].

### Dual luciferase reporter gene assay

The binding sites between lncRNA MAFG-AS1 and miR-149-3p and between HOXB8 and miR-149-3p were analyzed on a biological prediction website (https://cm.jefferson.edu/rna22/Interactive/), and the prediction results were further verified by dual luciferase reporter gene assay. Based on potential binding sequence of lncRNA MAFG-AS1 on miR-149-3p and the sequence on the 3′untranslated region (3′UTR) HOXB8 which miR-149-3p might bind to, target sequences and mutant (MUT) sequences were designed and constructed. Enzyme digestion was followed on pmiR-RB-REPORT™ plasmid (Ribobio Co., Ltd., Guangzhou, Guangdong, China) using restriction enzyme. Then, the artificially synthesized target gene fragments of wild type (WT) and MUT were inserted into pmiR-RB-REPORT™ plasmid (Ribobio) with miR-149-3p, respectively. Meanwhile, blank plasmids were transduced as a control group. The luciferase reporter plasmids WT and MUT following correctly sequencing were used for subsequent transfection. The cells were collected and lysed 48 h after transfection. The supernatant was obtained through centrifugation for 3–5 min. The luciferase activities of Renilla and firefly were measured in accordance with the manipulation method provided by the luciferase detection kit (RG005, Shanghai Beyotime Biotech Co., Ltd., Shanghai, China) with Renilla luciferase activity serving as the internal reference. The luminescent signal reflecting the activation of the target reporter gene was compared based on the ratio of the firefly relative light units (RLU) to the Renilla RLU value [20].

### Fluorescence in situ hybridization (FISH) assay

A lncRNA subcellular localization website (https://lncatlas.crg.eu/) was used to predict the expression and localization of lncRNA MAFG-AS1, which were further verified by FISH Kit (Roche, Basel, Switzerland). After transfection, HCT116 cells from each group were washed 2 times with cold phosphate buffered saline (PBS) and fixed in 4% paraformaldehyde. Then, hybridization solution containing digoxin labeled lncRNA MAFG-AS1 probes (Sigma-Aldrich Chemical Company, St Louis, MO, USA) was added to the cells. The antagonistic lncRNA MAFG-AS1 probes were considered as the NC. The cytoplasm was stained with 4-diamidine-2-phenylindole (Sigma-Aldrich) at room temperature for 10 min. After that, the cells were washed 2 times with cold PBS and the fluorescence images were observed and recorded under a confocal laser scanning microscope (FV1000, Olympus, Tokyo, Japan) [8].

### RNA immunoprecipitation (RIP) assay

The binding of lncRNA MAFG-AS1 and miR-149-3p to Argonaute-2 (Ago2) protein was analyzed by a RIP kit (Millipore, Billerica, MA, USA). The cells were rinsed with cold PBS, followed by the removal of the supernatant. RIPA lysis (P0013B, Beyotime) of equal volume was added for lysis in ice bath for 5 min. The supernatant was obtained through centrifugation at 14,000 rpm for 10 min at 4 °C. Cell extracts were divided into two parts; one for input, and the other was incubated with antibody for coprecipitation. Samples were placed in magnetic support for collecting the mixture of magnetic beads and protein. After protease K digestion, RNA was extracted from both samples and input for RT-qPCR. The antibody that were used for RIP were as follows: rabbit anti-human antibody to Ago2 (ab186733, 1:50, Abcam) for 30 min at room temperature and IgG (ab109489, 1:100, Abcam) as NC [21].

### Cell counting kit-8 (CCK-8) assay

Cell viability was determined by CCK-8 Kit (Solarbio). HCT116 and LoVo cells were seeded in a 96-well plate (ExcellBio, Taicang, Jiangsu, China) at a density of 1 × 10^4^ cells/well and cultured for 24 h. Then, 10 mL CCK-8 solution was added into each well. Cells were allowed to stand at 37 °C with 5% CO_2_ for 1–2 h. The optical density value was measured at 450 nm wavelength of the spectrophotometer [22].

### Transwell assay

Then, 48 h after transfection, cells were detached after 24-h starvation in the serum-free medium, washed 2 times by PBS and re-suspended in serum-free Opti-MEMI medium containing 10 g/mL BSA (31985008, NanJing SenBeiJia Biotechnology Co., Ltd., Jiangsu, China) to reach a density of 3 × 10^4^ cells/mL. Transwell chamber was placed on a 24-well plate, and the apical chamber of the bottom membrane of Transwell chamber was coated with Matrigel (40111ES08, Yeasen Biological Technology Co., Ltd., Shanghai, China) (diluted at 1:8) and air-dried at room temperature. Following conventional detachment and 2 PBS washes, the cells were re-suspended in RPMI 1640 medium at a density of 1 × 10^5^ cells/mL. Then, 200 μL cell suspension was added to the Matrigel-coated apical chamber, and 600 μL RPMI 1640 medium containing 20% FBS was added to the basolateral chamber. After 24-h culture, Transwell chamber was taken out and the cells on the interior surface of the apical chamber were removed by cotton swab. Later, the cells from the apical chamber were fixed in 4% polyformaldehyde for 15 min, stained with 0.5% crystal violet solution (prepared in methanol) for 15 min and washed 3 times with PBS. Five fields (× 200) were randomly selected under the inverted microscope (XDS-800D, Caikon Optical Instrument Co., Ltd., Shanghai, China). The number of cells that crossed the chamber membrane was counted [7].

### Flow cytometry

Following 48-h transfection, cells were detached by trypsin without ethylenediaminetetracetic acid and collected in a flow tube. After centrifugation and removal of the supernatant, the cells were washed 3 times by cold PBS, followed by another round of centrifugation to remove the supernatant. In accordance with the instructions of the Annexin-V-fluorescein isothiocyante (FITC) cell apoptosis detection kit (Sigma-Aldrich), Annexin-V-FITC, propidium iodide (PI) and N-2-hydroxyethylpiperazine-N-ethane-sulphonicacid (HEPES) buffer were mixed to produce Annexin-V-FITC/PI dye liquid in a proportion of 1:2:50. After that, 1 × 10^6^ cells were resuspended in 100 μL dye liquid and incubated at room temperature for 15 min, followed by addition of 1 mL HEPES, oscillated and mixed. Cell apoptosis was assessed by detecting fluorescence of FITC and PI using 525 and 620 nm bandpass filter at 480 nm wavelength [23].

### Lentiviral plasmid construction, packaging and transduction

A pLVX-Puro lentiviral vector (Clontech, Palo Alto, CA, USA) was used for the present study. The sequences of the shRNA cut sites of KpnI and XholI were selected and sent to Sangon Biotech Co., Ltd. (Shanghai, China) for primer synthesis. The PCR product was diluted 100-fold, and 1 µL of the PCR product was ligated with 200 ng linear plv-puro plasmid under T4 DNA ligase (NEB, Hertfordshire, UK) at room temperature for 2 h. The ligation products were transformed, amplified, and digested and identified after sequencing.

HEK293-FT cells (purchased from Chinese Academy of Sciences, Shanghai, China) in the logarithmic growth phase were seeded in petri dishes. After the cells were adhered to the wells, 7.5 µg psPAX2, 2.5 µg pMD2.G (Addgene plasmids 12259 and 12260, Abcam, Cambridge, MA, USA) and 10 µg of pLVX-RP1-93H18.6 were incubated with 100 µL of serum-free medium for 5 min. ViaFect ™ transfection reagent (Promega, Madison, WI, USA) was added, mixed with the lentiviral plasmid in a 3:1 volume ratio, and incubated for 20 min. The mixed solution was then added dropwise to the cells, and the supernatant containing virus particles was collected 48 h later. The cells were centrifuged at 2000 × *g* for 10 min at 4 °C, filtered and aliquoted, and stored at −80 °C.

HCT116 cells at exponential phase were transduced with empty lentiviral vector (sh-NC group), lentiviral shRNA targeting MAFG-AS1 (sh-MAFG-AS1), lentiviral mimic NC (mimic NC group), lentiviral miR-149-3p mimic (miR-149-3p mimic group), lentiviral sh-MAFG-AS1 and inhibitor NC (sh-MAFG-AS1 + inhibitor NC group) and lentiviral sh-MAFG-AS1 and miR-149-3p inhibitor (sh-MAFG-AS1 + miR-149-3p inhibitor). Cells were plated in six-well plates. When cell confluence reached 50% after 24 h, cells were added to Polybrene (Sigma, St. Louis, Mo., USA, final concentration = 8 μg/mL) and incubated in a cell incubator for 30 min. Next, 200 μL of virus was added to the cells in each well, and fresh medium was changed after 24 h of infection. After infection for 48 h, cells were sorted by puromycin (Sigma, St. Louis, Mo. final concentration 4 μg/mL). After sorting for about 10 days, cell lines with stable overexpression or knockdown of factors of interest were identified and stored frozen for future use [24].

### Tumor xenograft in nude mice

A total of 70 BALB/c nude mice (36 for HCT116 cells and 36 for LoVo cells), aged 4–6 weeks and weighed 14–18 g, were purchased from the Affiliated Hospital of Harbin Medical University (Harbin, Heilongjiang, China). The nude mice were raised in the animal experiment center of Southern Medical University before the experiment for 7 days under specific pathogen free (SPF) environment at suitable temperature with sterile feeding and drinking water under 12-h day/night cycle. HCT116 cells or LoVo cells transduced with different plasmids (n = 6 mice per group) in logarithmic growth phase and in good growth condition were suspended at a density of 5 × 10^7^ cells/mL. Next, 0.2 mL cell suspension was inoculated into the hypodermic side of the left fossa of each nude mice with a 1 mL syringe. After inoculation, the nude mice were raised under the laminar cover in the SPF animal room and observed on a regular basis. The short diameter (a) and long diameter (b) of tumors were recorded with Vernier calipers on the 7th, 14th, 21st and 28th day after inoculation. The tumor volume was calculated using the formula of π (a^2^b)/6 and the data were recorded. The nude mice were euthanized on the 28th day. The xenografted tumors of nude mice were resected under the aseptic condition, fixed in 4% paraformaldehyde for 24 h, embedded with paraffin and fixed [25].

### Immunohistochemistry (IHC)

The paraffin blocks of human CRC and adjacent tissues and xenografted tumors from nude mice in each group were sliced into 4 μm sections using a slice machine (RM2016, Leica Co., Ltd., Shanghai, China). Then, the sections were attached to the slide glass treated by polylysine, baked for 4.5 h at 65 °C, dewaxed by xylene (2 times, 30 min each time), and then dehydrated in gradient alcohol of 100, 95, 80, and 70% respectively (5 min each time) and washed by running water for 2 min. Next, the sections were immersed in 3% methanol H_2_O_2_ for 20 min and then washed by distilled water for 2 min and by 0.1 M PBS for 3 min. Then, antigen repair was performed in a water bath. The sections were blocked with normal goat serum sealing fluid (C-0005, Haoran Biological Technology Co., Ltd., Shanghai, China) at room temperature for 20 min, probed with rabbit anti-mouse primary antibody to HOXB8 (PA5-81200, 1:100, Invitrogen) overnight at 4 °C, and re-probed with goat anti-rabbit secondary antibody to IgG (1:1000, ab6785, Abcam) at 37 °C for 20 min. After that, the enzyme-labeled streptavidin ovalbumin working solution (Bio-High Technology Co., Ltd., Hebei, China) was added and incubated with the sections at 37 °C for 20 min. Later, the sections were developed by 3,3′-Diaminobenzidine (Bio-High), counterstained for 1 min with hematoxylin, immersed in 1% ammonia to return blue in color, dehydrated by alcohol of gradient concentrations, cleared by xylene, and mounted by neutral resin. The staining results were observed under the microscope and photographs of the sections were taken. In each section, 5 high magnification fields were randomly selected with 100 cells in each field. The ratio of positively stained cells in each section < 10% was considered as negative, the ratio of positively stained cells between 10 and 50% was regarded as positive, and the ratio of positively stained cells > 50% was thought as strongly positive [26].

### Statistical analysis

All data were analyzed with SPSS 21.0 statistical software (IBM, Armonk, NY, USA). The test of normal distribution and homogeneity of variance was carried out. The measurement data in normal distribution were expressed by mean ± standard deviation. The comparison between tumor and adjacent tissues was conducted by paired *t* test while data comparison between other two groups was performed by unpaired *t* test, and the comparison among multiple groups by one-way analysis of variance (ANOVA). The pairwise comparison was analyzed by Tukey’s post hoc test. The data at different time points were analyzed by repeated measures ANOVA, followed by Bonferroni test. All skewed distribution data were tested by nonparametric rank sum test. A value of *p* < 0.05 was considered with significantly statistical difference.

## Results

### LncRNA MAFG-AS1 was highly expressed and miR-149-3p was poorly expressed in CRC tissues and cells

**Fig. 1:**
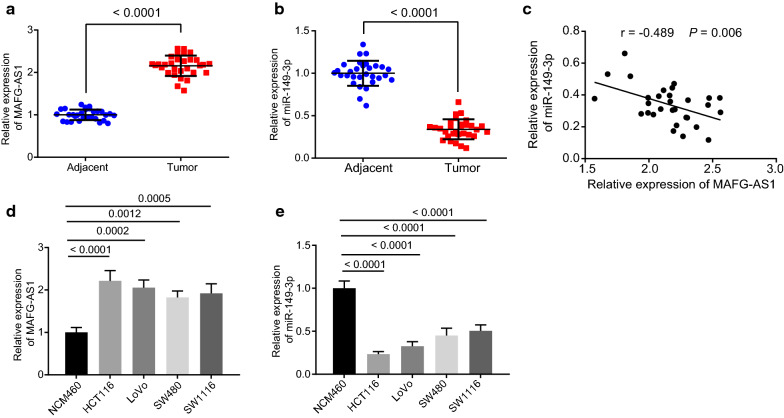
High expression of lncRNA MAFG-AS1 and low expression of miR-149-3p are observed in CRC tissues and cells. **a** The expression of lncRNA MAFG-AS1 in CRC tissues and adjacent tissues (n = 30). **b** The expression of miR-149-3p in CRC tissues and adjacent tissues (n = 30). **c** The correlation analysis on the expression of lncRNA MAFG-AS1 and miR-149-3p. **d** The expression of lncRNA MAFG-AS1 in CRC cell lines (HCT116, LoVo, SW480 and SW1116) and normal colon epithelial cell line NCM460 determined by RT-qPCR. **e** The expression of miR-149-3p in CRC cell lines (HCT116, LoVo, SW480 and SW1116) and normal colon epithelial cell line NCM460 determined by RT-qPCR. * *p* < 0.05 *vs.* the adjacent tissues or NCM460 cell line. Data between two groups were compared by paired *t* test. The correlation analysis between expression of lncRNA MAFG-AS1 and miR-149-3p was performed using Pearson Correlation Coefficient. Data among multiple groups were compared by one-way ANOVA, followed by Tukey’s post hoc test. The experiment was repeated 3 times independently

Initially, to explore the regulatory mechanism of lncRNA MAFG-AS1 and miR-149-3p in CRC, RT-qPCR was performed to determine expression pattern of lncRNA MAFG-AS1 and miR-149-3p in 30 clinically obtained CRC tissues and adjacent tissues. Results revealed significantly upregulated lncRNA MAFG-AS1 (*p* < 0.0001, Fig. 1a) and downregulated miR-149-3p (*p* < 0.0001, Fig. 1b) in CRC tissues than those in adjacent tissues. Correlation analysis between lncRNA MAFG-AS1 and miR-149-3p in CRC tissues showed a negative relationship (Fig. 1c). Further exploration was performed considering the expression pattern of lncRNA MAFG-AS1 and miR-149-3p in CRC cell lines, HCT116, LoVo, SW480 and SW1116, and normal colon epithelial cell line NCM460. Consistently, upregulated lncRNA MAFG-AS1 (Fig. 1d) and downregulated miR-149-3p (Fig. 1e) were observed in CRC cell lines than those in NCM460 cell line. Taken together, these findings demonstrated high expression of lncRNA MAFG-AS1 and low expression of miR-149-3p in CRC tissues and cells.

### Silencing lncRNA MAFG-AS1 or overexpressed miR-149-3p inhibited CRC cell proliferation, migration and invasion

**Fig. 2:**
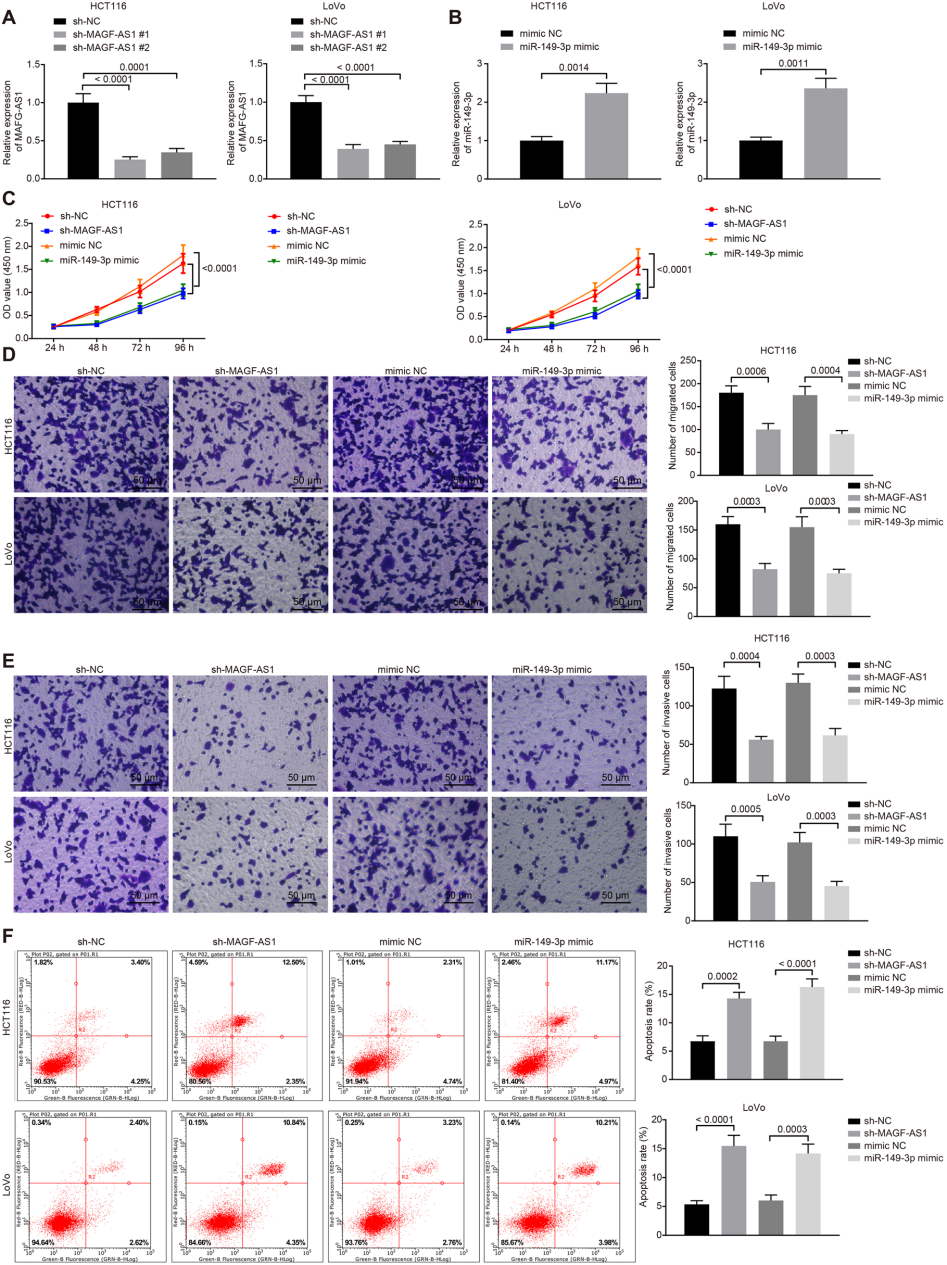
Silence of lncRNA MAFG-AS1 or upregulation of miR-149-3p exerts inhibitory effects on proliferation, migration and invasion of CRC cells. **a** The expression of lncRNA MAFG-AS1 in HCT116 and LoVo cells determined by RT-qPCR. **b** The expression of miR-149-3p in HCT116 and LoVo cells determined by RT-qPCR. **c** The HCT116 and LoVo cell proliferation detected by CCK-8 assay. **d** The HCT116 and LoVo cell migration detected by Transwell assay (scale bar = 50 µm). **e** The HCT116 and LoVo cell invasion detected by Transwell assay (scale bar = 50 µm). **f** The HCT116 and LoVo cell apoptosis detected by flow cytometry. Data between two groups were compared by unpaired *t* test. Data among multiple groups were compared by one-way ANOVA, followed by Tukey’s post hoc test. Data at different time points were analyzed by repeated measures ANOVA, followed by Bonferroni test. The experiment was repeated 3 times independently

With results determining the aberrantly expressed lncRNA MAFG-AS1 and miR-149-3p in CRC tissues and cells, the research focus was shifted to the biological function of lncRNA MAFG-AS1 and miR-149-3p in CRC cells. LncRNA MAFG-AS1 was silenced and miR-149-3p was overexpressed in HCT116 and LoVo cell lines, and the transfection efficiency was detected by RT-qPCR. Results showed that the delivery of sh-MAFG-AS1#1 led to much lower expression of lncRNA MAFG-AS1 both in HCT116 and LoVo cell lines (Fig. 2a). Therefore, sh-MAFG-AS1#1 was selected for subsequent experiments. Besides, delivery of miR-149-3p mimic resulted in significantly high expression of miR-149-3p both in HCT116 and LoVo cell lines (Fig. 2b), suggesting that miR-149-3p mimic could be used for following experiments. Subsequently, cell proliferation, migration and invasion and apoptosis were assessed by CCK-8 assay (Fig. 2c), Transwell assay (Fig. 2d, e) and flow cytometry (Fig. 2f), respectively. It was found that cell proliferation, migration and invasion were significantly suppressed while cell apoptosis was promoted both in HCT116 and LoVo cell lines in the presence of silenced lncRNA MAFG-AS1 or overexpressed miR-149-3p. To conclude, CRC cell malignant behaviors could be inhibited by downregulating lncRNA MAFG-AS1 or upregulating miR-149-3p.

### LncRNA MAFG-AS1 could bind to miR-149-3p and downregulate miR-149-3p expression

**Fig. 3:**
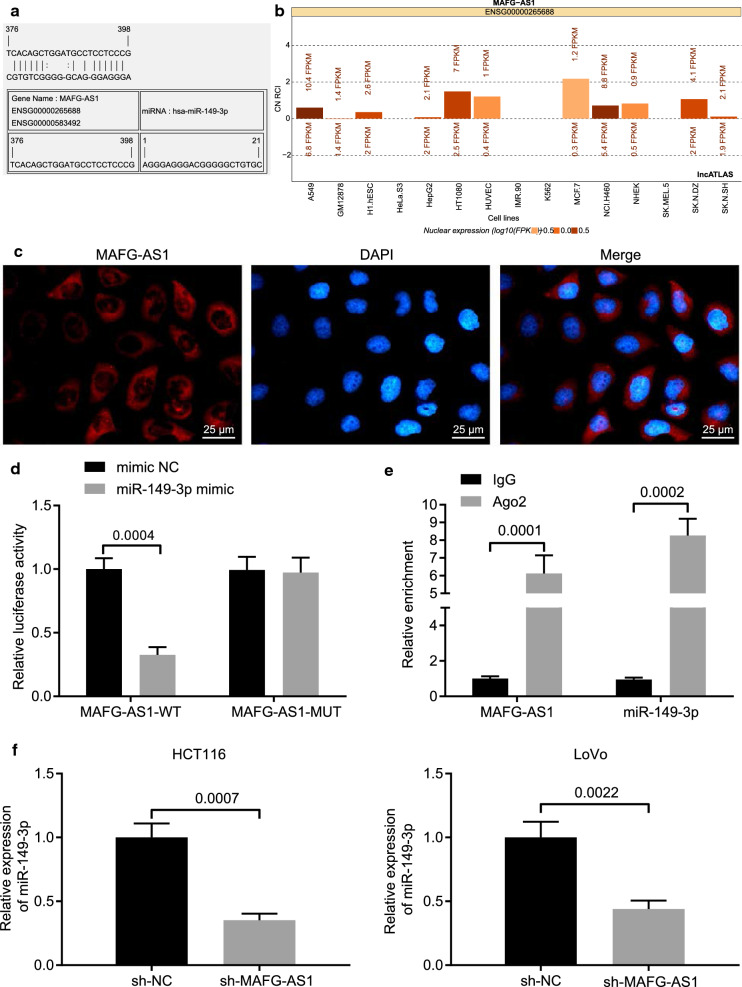
LncRNA MAFG-AS1 specifically binds to miR-149-3p and downregulates miR-149-3p expression. **a** The predicative binding sites between miR-149-3p and lncRNA MAFG-AS1. **b** The subcellular localization of lncRNA MAFG-AS1 predicted online. **c** The expression of lncRNA MAFG-AS1 in HCT116 cells detected by FISH assay (scale bar = 25 µm). **d** The binding between lncRNA MAFG-AS1 and miR-149-3p detected by dual luciferase reporter gene assay. **e** The binding of lncRNA MAFG-AS1 and miR-149-3p to Ago2 detected by RIP assay. **f** The expression of miR-149-3p in HCT116 and LoVo cells determined by RT-qPCR. Data between two groups were compared by unpaired *t* test. The experiment was repeated 3 times independently

To further investigate the mechanism of lncRNA MAFG-AS1 and miR-149-3p in CRC, online analysis software was utilized to predict the relationship between lncRNA MAFG-AS1 and miR-149-3p, the results of which revealed specific binding sites between lncRNA MAFG-AS1 and miR-149-3p (Fig. 3a). The subcellular localization of lncRNA MAFG-AS1 was further predicted online (https://lncatlas.crg.eu). Results suggested that lncRNA MAFG-AS1 was mainly expressed in the cytoplasm (Fig. 3b), which was further verified by FISH assay (Fig. 3c). Dual luciferase reporter gene assay was performed to detect the binding between lncRNA MAFG-AS1 and miR-149-3p (Fig. 3d), which revealed that compared with mimic NC, the luciferase activities of MAFG-AS1-WT could be significantly suppressed by miR-149-3p mimic (*p* < 0.05).

RIP assay was conducted to detect the binding of lncRNA MAFG-AS1 and miR-149-3p to Ago2, and results showed that enrichment of lncRNA MAFG-AS1 and miR-149-3p in Ago2 precipitate was significantly higher relative to IgG (Fig. 3e). Therefore, lncRNA MAFG-AS1 might bind to miR-149-3p. The miR-149-3p expression in response to silencing lncRNA MAFG-AS1 was then determined by RT-qPCR, and results revealed significantly elevated miR-149-3p expression in the presence of sh-MAFG-AS1 in comparison to sh-NC (Fig. 3f). To sum up, these findings elucidated that lncRNA MAFG-AS1 could bind to miR-149-3p and negatively regulate the expression of miR-149-3p.

### LncRNA MAFG-AS1 promoted HOXB8 expression via miR-149-3p

**Fig. 4:**
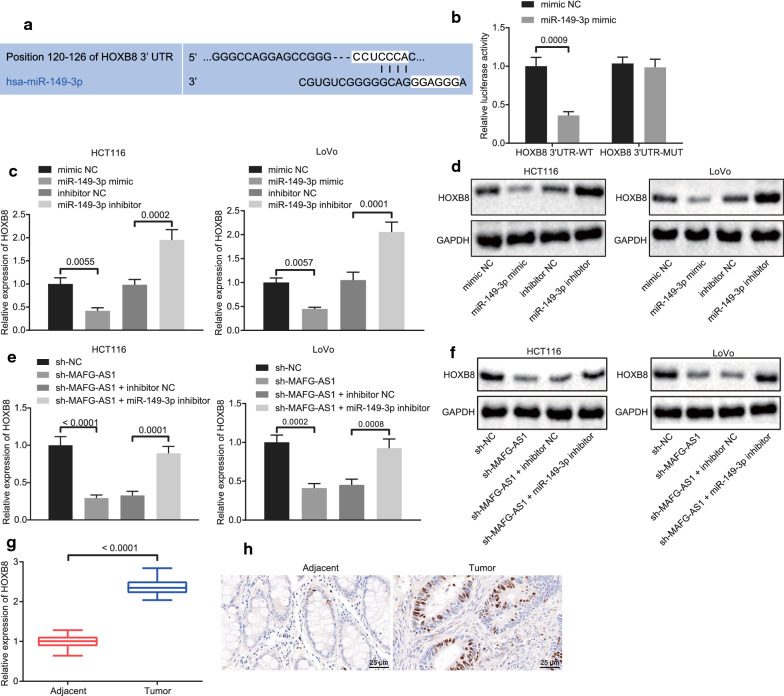
LncRNA MAFG-AS1 regulates the expression HOXB8 by targeting miR-149-3p. **a** The binding sites between miR-149-3p and HOXB8 predicted by Targetscan, which is an online prediction tool for biological targets of miRNA by identifying conservative 8mer, 7mer and 6mer sites that match the seed region of each miRNA. **b** The binding between miR-149-3p and HOXB8 detected by dual luciferase reporter gene assay. **c** The mRNA expression of HOXB8 in response to different expression patterns of miR-149-3p in HCT116 and LoVo cells determined by RT-qPCR. **d** The protein expression of HOXB8 in response to different expression patterns of miR-149-3p normalized to GAPDH in HCT116 and LoVo cells determined by Western blot analysis. **e** The mRNA expression of HOXB8 in response to different expression patterns of lncRNA MAFG-AS1 and miR-149-3p in HCT116 and LoVo cells determined by RT-qPCR. **f** The protein expression of HOXB8 in response to different expression patterns of lncRNA MAFG-AS1 and miR-149-3p normalized to GAPDH in HCT116 and LoVo cells determined by Western blot analysis. **g** The expression of HOXB8 in CRC tissues and adjacent tissues determined by RT-qPCR (n = 30). **h** The expression of HOXB8 in CRC tissues and adjacent tissues detected by immunohistochemistry (scale bar = 25 µm). Data between two groups were compared by unpaired *t* test but by paired *t* test between CRC tissues and adjacent tissues. Data among multiple groups were compared by one-way ANOVA, followed by Tukey’s post hoc test. The experiment was repeated 3 times independently

The downstream molecular mechanism of miR-149-3p was further explored. Potential binding sites between miR-149-3p and HOXB8 were predicted through the miRNA target prediction tool Targetscan website (https://www.targetscan.org/vert_72/) (Fig. 4a). Dual luciferase reporter gene assay for binding detection between miR-149-3p and HOXB8 showed that miR-149-3p mimic significantly inhibited the luciferase activity of HOXB8 3′UTR when compared with mimic NC (Fig. 4b). The mRNA and protein expression of HOXB8 was assessed by RT-qPCR (Fig. 4c) and Western blot analysis (Fig. 4d), respectively, following treatment of miR-149-3p inhibitor or miR-149-3p mimic. Results showed that mRNA and protein expression of HOXB8 was significantly elevated in response to miR-149-3p inhibitor but diminished in response to miR-149-3p mimic.

Furthermore, lncRNA MAFG-AS1 was silenced both in HCT116 and LoVo cells where HOXB8 mRNA and protein expression was significantly reduced as assessed by RT-qPCR (Fig. 4e) and Western blot analysis (Fig. 4f). In the presence of silenced lncRNA MAFG-AS1, miR-149-3p inhibitor was delivered, by which mRNA and protein expression of HOXB8 was significantly restored. The expression pattern of HOXB8 in CRC tissues determined by RT-qPCR turned out to be upregulated compared with adjacent tissues (Fig. 4g), which was further verified by immunohistochemistry (Fig. 4h). The above results highlighted that lncRNA MAFG-AS1 played a mediatory role in the progression of CRC by upregulating the expression of HOXB8 through miR-149-3p.

### LncRNA MAFG-AS1 upregulated HOXB8 via miR-149-3p leading to CRC cell proliferation, migration and invasion

**Fig. 5:**
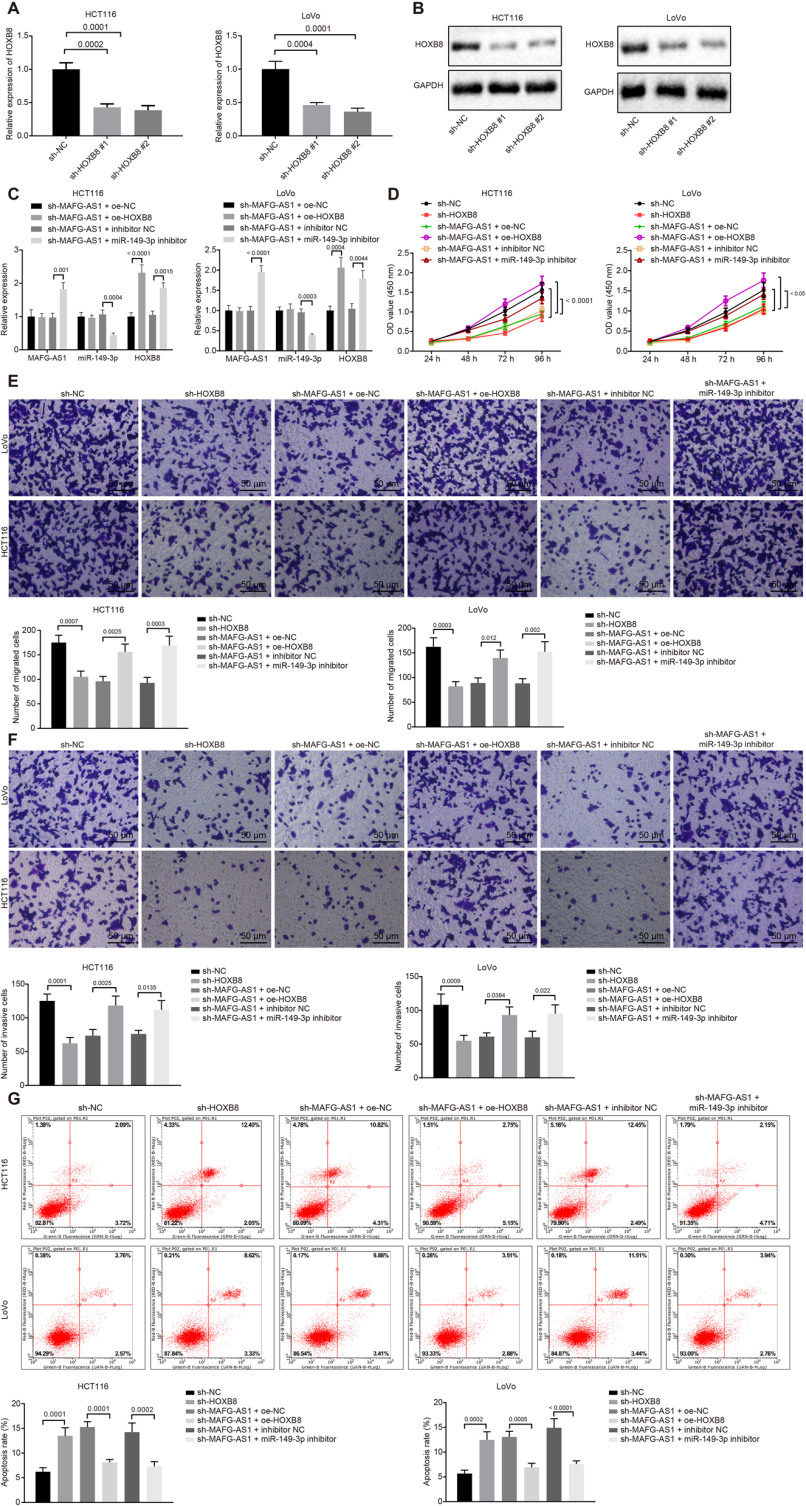
The lncRNA MAFG-AS1/miR-149-3p/HOXB8 axis promotes proliferation, migration and invasion of CRC cells in vitro. **a** The mRNA expression of HOXB8 in HCT116 and LoVo cells determined by RT-qPCR. **b** The protein expression of HOXB8 in HCT116 and LoVo cells normalized to GAPDH determined by Western blot analysis. **c** The expression of lncRNA MAFG-AS1, miR-149-3p and HOXB8 in HCT116 and LoVo cells determined by RT-qPCR. **d** The HCT116 and LoVo cell proliferation detected by CCK-8 assay. **e** The HCT116 and LoVo cell migration detected by Transwell assay (scale bar = 50 µm). **f** The HCT116 and LoVo cell invasion detected by Transwell assay (scale bar = 50 µm). **g** The HCT116 and LoVo cell apoptosis detected by flow cytometry. Data among multiple groups were compared by one-way ANOVA, followed by Tukey’s post hoc test. Data at different time points were analyzed by repeated measures ANOVA, followed by Bonferroni test. The experiment was repeated 3 times independently

Subsequently, HCT116 and LoVo cells were treated with silenced HOXB8, and RT-qPCR and Western blot analysis were applied to detect the silencing efficiency (Fig. 5a, b). Results showed that both sh-HOXB8#1 and sh-HOXB8#2 led to significantly downregulated HOXB8 while HOXB8 expression was much lower in the presence of sh-HOXB8#2, which was selected for following experiments.

Gain- and loss-of-function assays were performed to explore the effects of the lncRNA MAFG-AS1/miR-149-3p/HOXB8 axis in the biological functions of CRC cells by co-silence of lncRNA MAFG-AS1 and miR-149-3p as well as by silence of lncRNA MAFG-AS1 together with overexpression of HOXB8. RT-qPCR for quantification analysis showed that in the presence of sh-MAFG-AS1 + oe-HOXB8, expression of lncRNA MAFG-AS1 and miR-149-3p remained unchanged while HOXB8 expression significantly increased. In response to sh-MAFG-AS1 + miR-149-3p inhibitor, lncRNA MAFG-AS1 was upregulated, miR-149-3p was downregulated and HOXB8 expression was restored (Fig. 5c).

Furthermore, cell proliferation, migration and invasion and apoptosis were assessed by CCK-8 assay (Fig. 5d), Transwell assay (Fig. 5e, f) and flow cytometry (Fig. 5g), respectively. Compared with sh-NC treatment, the cell proliferation in the presence of sh-HOXB8 treatment was reduced (*p* = 0.0284). Compared with sh-MAFG-AS1 + oe-NC treatment, sh-MAFG-AS1 + oe-HOXB8 resulted in increased cell proliferation (*p* = 0.0086). Compared with sh-MAFG-AS1 + inhibitor NC treatment, sh-MAFG-AS1 + miR-149-3p inhibitor resulted in recovery of cell proliferation (*p* = 0.0411) (Fig. 5d). It was observed that sh-HOXB8 resulted in significantly weakened cell proliferation, migration and invasion along with enhanced apoptosis. Based on the presence of sh-MAFG-AS1, opposite changing tendency was found with delivery of oe-HOXB8 while miR-149-3p inhibitor significantly restored the cell capacities of proliferation, migration, invasion, and apoptosis. Taken together, our experimental data indicated that the lncRNA MAFG-AS1/miR-149-3p/HOXB8 axis contributed to the progression of CRC.

### LncRNA MAFG-AS1 upregulated HOXB8 via miR-149-3p in leading to CRC cell tumorigenicity in vivo

**Fig. 6:**
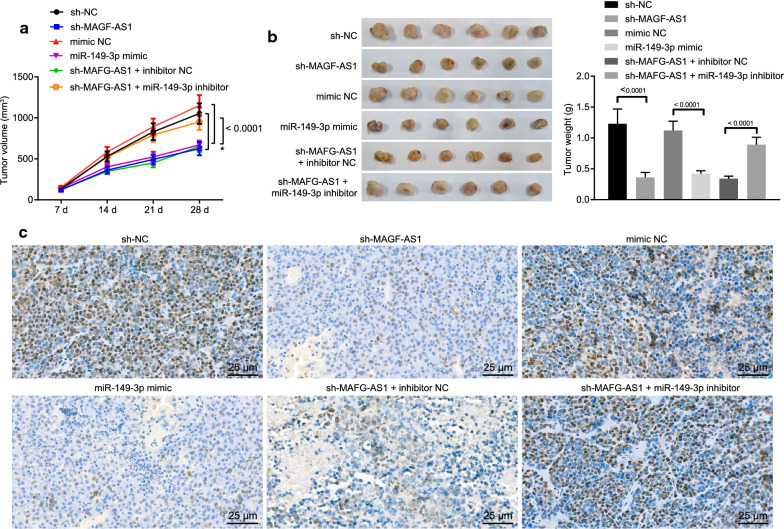
The lncRNA MAFG-AS1/miR-149-3p/HOXB8 axis promotes tumorigenesis of CRC cells (HCT116 cells) in vivo. **a** Tumor volume from the 7th day after injection. **b** Representative pictures of resected tumors and tumor weight. **c** The expression of HOXB8 in tumors identified by immunohistochemistry (scale bar = 25 µm). Data among multiple groups were compared by one-way ANOVA, followed by Tukey’s post hoc test. Data at different time points were analyzed by repeated measures ANOVA, followed by Bonferroni test. The experiment was repeated 3 times independently

At last, the tumor xenograft experiments in nude mice were carried out for the detection of tumorigenesis of CRC cells involving the lncRNA MAFG-AS1/miR-149-3p/HOXB8 axis in vivo by subcutaneous injection of HCT116 cells (Fig. 6) and LoVo cells (Additional file 1: Figure S1) harboring sh-NC, sh-MAFG-AS1, mimic NC, miR-149-3p mimic, sh-MAFG-AS1 + inhibitor NC or sh-MAFG-AS1 + miR-149-3p inhibitor. The tumor volume (Fig. 6a, Additional file 1: Figure S1A) and weight (Fig. 6b, Additional file 1: Figure S1B) were measured from the 7th day after injection. Results showed that tumor volume and weight were significantly smaller in the presence of sh-MAFG-AS1 or miR-149-3p mimic but restored in the presence of sh-MAFG-AS1 + miR-149-3p inhibitor in comparison to their corresponding controls. The expression of HOXB8 in tumors from each group was detected by immunohistochemistry (Fig. 6c, Additional file 1: Figure S1C). Results showed that HOXB8 expression was reduced by sh-MAFG-AS1 or miR-149-3p mimic and restored by sh-MAFG-AS1 + miR-149-3p inhibitor. In agreement with these observations, we could reach a conclusion that the lncRNA MAFG-AS1/miR-149-3p/HOXB8 axis contributed to the tumorigenicity of CRC cells in vivo.

## Discussion

As one of the most fetal diseases, CRC usually becomes symptomic at an advanced stage so that the development of effective early detection techniques is imperative [27]. Accumulating evidences have focused on the action of various lncRNAs in CRC through their interaction with different miRNAs [28–30]. The current study was carried out exploring the role of the lncRNA MAFG-AS1/miR-149-3p/HOXB8 functional axis in CRC with the aim to offer new insights into the early detection, diagnosis and treatment modes of CRC. The experimental data in our study proved that silencing lncRNA MAFG-AS1 or overexpressing miR-149-3p could curb proliferation, invasion, migration and tumorigenicity, but promote apoptosis of CRC cells via the inhibition of HOXB8 expression.

Based on our findings, lncRNA MAFG-AS1 and HOXB8 were found to exhibit high levels of expression in CRC tissues and cells where miR-149-3p was expressed at a low level. According to a regulatory network analysis of lncRNAs in CRC, lncRNA MAFG-AS1 has been revealed as an upregulated lncRNA with strong regulatory functions [31]. In line with our finding, expression of lncRNA MAFG-AS1 has been reported to be higher in CRC tissues in association with advanced tumor stage and enhanced cell proliferation and invasion as well as suppressed apoptosis [11]. Besides, the delivery of miR-149-3p mimic has been indicated to promote HCT116 cell apoptosis while downregulation of miR-149-3p negatively impacts on chemotherapeutic effects induced by fluorouracil [14]. By targeting Forkhead Box M1, low expression of miR-149 has been observed to harness the prognostic potential in parallel with unsatisfactory oncologic outcomes of patients with CRC [32]. Consistently, HOXB8 expression has been found to be elevated in CRC tissues while inhibition of HOXB8 is associated with enhanced sensitivity of CRC cells to chemotherapy [33]. Silence of HOXB8 has been unraveled to reverse transcriptional signatures related to malignant phenotypes in CRC cells [34]. Give the aforementioned evidence, the gain- and loss-of-function assays in our study were supported that silencing lncRNA MAFG-AS1 or HOXB8 harbored the potential to inhibit the proliferation, invasion, migration and tumorigenicity but promote the apoptosis of CRC cells, all of which were also observed in response to upregulation of miR-149-3p.

Further regulatory mechanism exploration in our study demonstrated that lncRNA MAFG-AS1 could target and negatively regulate miR-149-3p and miR-149-3p could further bind to HOXB8 while lncRNA MAFG-AS1 functioned in CRC by mediating the expression of HOXB8 via miR-149-3p. The interactions among mRNA, miRNAs and lncRNAs in CRC have been proposed with great therapeutic potential from perspectives of tumorigenesis, tumor growth and progression for development on new effective biomarkers of diagnosis, treatment and prognosis [35, 36]. As shown in a previous study reporting the oncogenic role of lncRNA MAFG-AS1 in CRC, miR-147b and NDUFA4 function as interactors with lncRNA MAFG-AS1 in a similar manner [11]. Additionally, lncRNA MAFG-AS1 has been elaborated to interact with miR-339-5p and matrix metalloproteinase 15, facilitating the aggressive phenotypes of breast cancer and non-small-cell lung cancer [9, 10]. In the setting of breast cancer, lncRNA Migration Inhibitory Factor Antisense RNA 1, overexpressed in breast cancer tissues and cells, has been identified to boost the progression through regulation of HOXB8 by binding to miR-1249-3p [37]. Notably, downregulation of miR-149, targeted by lncRNA PCAT-1, has been documented to have the capacity of reversing the inhibitory effects of downregulated lncRNA PCAT-1 on CRC cell growth [15]. Likewise, our experimental data exhibited that the suppressive effects of lncRNA MAFG-AS1 on malignant biological characteristics of HCT116 and LoVo cells were also significantly abrogated in presence of miR-149-3p inhibitor.

## Conclusion

**Fig. 7:**
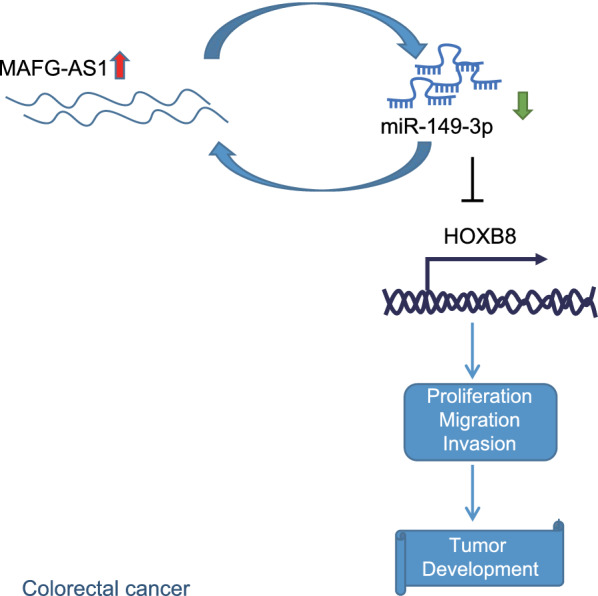
Mechanistic investigations indicated that lncRNA MAFG-AS1 could specifically bind to miR-149-3p and negatively regulate miR-149-3p expression to upregulate its downstream gene HOXB8, thereby promoting the proliferation, migration, invasion and tumorigenesis of CRC cells while inhibiting apoptosis

In conclusion, knockdown of lncRNA MAFG-AS1 or overexpression of miR-149-3p would contribute to inhibition of proliferation, invasion and migration but promotion of apoptosis of CRC cells. As displayed in Fig. 7, downregulation of lncRNA MAFG-AS1 promotes miR-149-3p expression to inhibit HOXB8, thereby inhibiting the proliferation, migration, invasion and tumorigenesis of CRC cells. To sum up, our finding identified the functional lncRNA MAFG-AS1/miR-149-3p/HOXB8 axis as a potential prognostic biomarker and therapeutic target in CRC. However, due to the limited currently available tools to elucidate lncRNA mechanisms, further investigations are required considering the accessibility of miRNAs and lncRNAs to excavate further validation of our results for therapeutic intervention in the clinical application.

## Supplementary information

**Additional file 1: Figure S1.** The lncRNA MAFG-AS1/miR-149-3p/HOXB8 axis promotes tumorigenesis of CRC cells (LoVo cells) in vivo. A, Tumor volume from the 7th day after injection. B, Representative pictures of resected tumors and tumor weight. C, The expression of HOXB8 in tumors identified by immunohistochemistry (scale bar = 25 µm). Data among multiple groups were compared by one-way ANOVA, followed by Tukey’s post hoc test. Data at different time points were analyzed by repeated measures ANOVA, followed by Bonferroni test. The experiment was repeated 3 times independently.

## Data Availability

Data generated and analyzed as part of this study are included in the manuscript or are available upon request from the corresponding author.

## Abbreviations

CRC: Colorectal cancer
lncRNAs: Long non-coding RNAs
MAFG-AS1: MAF BZIP Transcription Factor G Antisense RNA 1
miR-149-3p: MicroRNA-149-3p
HOXB8: Homeobox B8
PCAT-1: Prostate cancer-associated ncRNA transcripts 1
NC: Negative control
RT-qPCR: Reverse transcription quantitative polymerase chain reaction
GAPDH: Glyceraldehyde-3-phosphate dehydrogenase
RIPA: Radio-immunoprecipitation assay
IgG: Immunoglobulin G
BSA: Bovine serum albumin
3′UTR: 3′untranslated region
MUT: Mutant
WT: Wild type
FISH: Fluorescence in situ hybridization
RIP: RNA immunoprecipitation
CCK-8: Cell counting kit-8
assayFITC: Fluorescein isothiocyante
HEPES: n-2-hydroxyethylpiperazine-N-ethane-sulphonicacid
SPF: Specific pathogen free
IHC: Immunohistochemistry
ANOVA: Analysis of variance
